# Preparation, Diagnosis, Biological Activity, and Theoretical Studies of Some Mixed Drug Complexes

**DOI:** 10.1155/2019/8962923

**Published:** 2019-05-08

**Authors:** Bayader F. Abbas, Barakat A. F. Kamel, Wessal M. Khamais

**Affiliations:** Department of Chemistry, College of Science, Al-Mustansiriyah University, Baghdad, Iraq

## Abstract

This paper includes synthesis and characterization of mixed ligand complexes derived from mefenamic acid and metformin using transition metal ions such as Co(II) and Cu(II). These complexes have been characterized by magnetic susceptibility, molar conductance, TG analyses, and spectral techniques such as FTIR and UV spectra. The theoretical study of the ligands and their complexes using semiempirical (PM6) method was used to measure IR and UV spectroscopy, HOMO-LUMO categories of the ligands. These synthesized complexes are also studied for their biological activities. The studies made on these complexes proposed a six octahedral geometry.

## 1. Introduction

Mefenamic acid [L_1_] 2-[(2,3- dimethylphenyl)amino]benzoic acid], an anthranilic acid derivative, which is widely used to relieve mild to moderate pain [1], demonstrates nonsteroidal anti-inflammatory, antipyretic, and analgesic activity [2, 3]. Metformin [L_2_] has two amino groups that have an excellent ability to coordinate with many transitional chain elements, thus giving highly colored clonal complexes, especially copper (II), nickel (II), and cobalt (II), because these metal ion complexes can be measured spectrally [4–6]. Metformin is a low-cost antioxidant. Metformin tablets also help reduce body weight or at least weight stability. Most tablets are used to treat high blood sugar that causes increased body weight [7]. Metformin hydrochloride is freely soluble in water and practically soluble in acetone, ether, and chloroform, and pH of a 1% water solution of metformin hydrochloride is 6.68 [8]. All the synthesized mixed ligand complexes (0.5 mg/mL) were screened for their antibacterial activities against five bacteria* (Staphylococcus aureus, Escherichia coli, Streptococcus sp., Candida albicans,* and* Pseudomonas aeruginosa*) by agar well diffusion assay method [9] using DMSO as control. The standard Tetracycline (0.05 mg/mL) antibiotic was used as an antibacterial agent. The inoculated plates were incubated at 30°C and 37°C temperature for 48 hours, and inhibition zone was measured in mm. Theoretically the PM6 semiempirical method was used to carry out the calculations for the ligands and their complexes after being constructed and was used to analyze the structural and electronic parameters; the structures were fully optimized and vibrational analysis was carried out to verify that the optimized geometries corresponded to minimum global energy [10].

## 2. Materials and Methods

### 2.1. Materials

All the chemicals were supplied from Samarra Laboratories for Drugs Industry (IRAQ), BDH, and Fluka; these materials were used without further purification.

### 2.2. Instruments

Atomic absorption using Shimadzu model 6809, FTIR-8300 Shimadzu spectrophotometer, in the frequency range of 4000-400 cm^−1^, UV-visible spectrophotometer using Varian model, and conductivity meter using Philips. The melting points were recorded in Coslab melting point apparatus. The magnetic susceptibility of the solid complexes was obtained at room temperature using Magnetic Susceptibility Balance Johnson Matthey. Elemental analysis (EA) was carried out using elemental analyzer.

### 2.3. Methods

#### 2.3.1. Synthesis of Mixed Drug Complexes

**Scheme 1:**
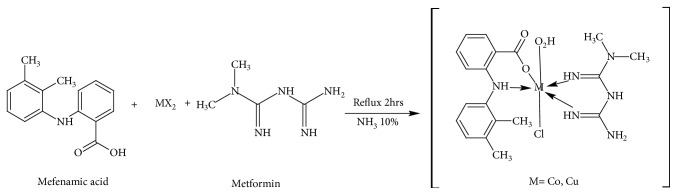
Synthesis of mixed drug complexes.

Synthesis of mixed drug complexes was carried out using template method. A hot ethanolic solution (10 ml) of respective transition metal salts (1 mmol) was mixed with a hot solution (1 mmol) of mefenamic acid and metformin (1 mmol). Few drops of dil. NH3 10% were added to the mixture. The resulting mixture was then left under reflux for 2 hours. After refluxing for 2hrs appropriate complexes were precipitated out on cooling the reaction mixture. Those were then filtered, washed with ethanol, and washed using cold water [11, 12]. The synthesized complexes were obtained in 65-72% yield.  Scheme 1 shows the mechanism for synthesis of the mixed drug complexes.

## 3. Results and Discussion

### 3.1. Physicochemical Properties of Ligands and Complexes

All the synthesized complexes were thermally stable and colored. The magnetic moments of the solid complexes were measured by Farady's method at 300 K and the values observed for copper(II) complex were 1.69-1.78 BM which fall in one of the expected electrons for d^9^ configurations [13]. However, the increased magnetic susceptibility of cobalt(II) complexes in the 4.75-4.85 BM region supports the octahedral geometry around Co(II) ion due to orbital contribution.  Table 1 shows the physicochemical properties of these synthesized complexes.

**Figure 1:**
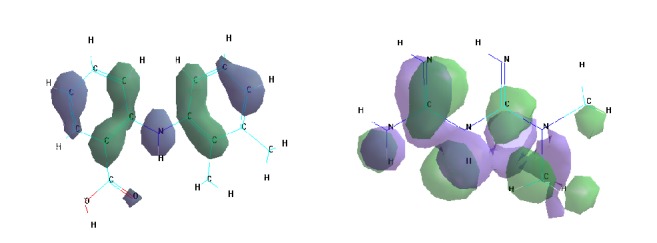
HOMO-LUMO categories of the ligands (mefenamic acid and metformin).

Figure 1 shows the HOMO-LUMO categories for the ligands according to semiempirical method (PM6).

### 3.2. IR Spectra of Ligands and Complexes

**Figure 2:**
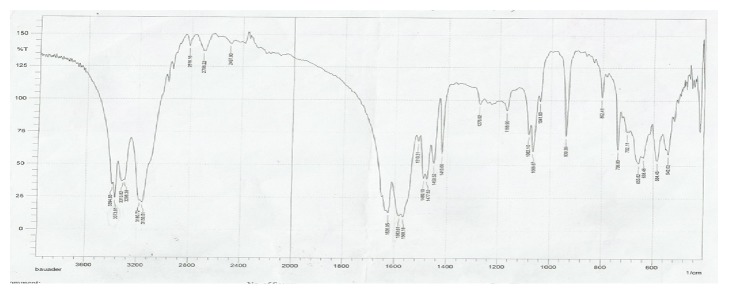
The IR spectra of the ligand metformin (L_2_).

**Figure 3:**
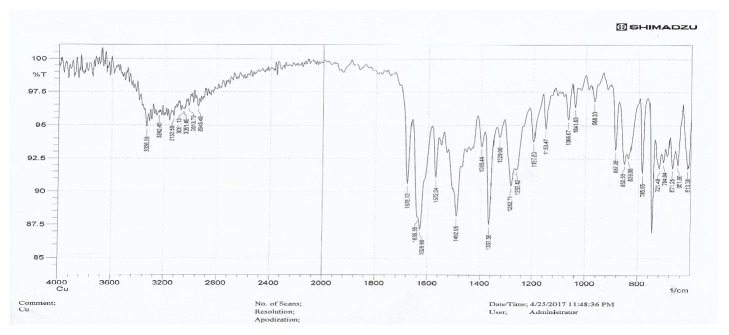
The IR spectra of the complex [Cu(L1)(L2) H_2_OCl].

The ligand (metformin) shows three sharp absorption bundles at regions 3373cm^−1^ and 3159-3298 cm^−1^ assigned to groups NH and NH_2_, respectively; peak C = N appears at 1583- 1626cm^−1^ and 1276-1419cm^−1^ assigned to C=N; and N-N appears at 937cm^−1^. Mefenamic acid has N-H band appearing at 3340cm^−1^. The vibrational spectrum of the synthesized complexes showed the difference in the severity of the packs C = N and NH, respectively, having clearer deviations than in the free ligand. This is evidence of the participation of the nitrogen atom in the uniformity of the metal ion. This is shown by resonance and the change in the intensity of the group C = N towards the red displacement in the complex was recorded in the free ligand (1583-1626cm^−1^) (1612-1629cm^−1^) in the metal complex due to consistency and the formation of the motor stabilized system [10]. The presence of 812cm^−1^ refers to the frequency of M-N=C group and the new band in the range of (486-449)cm^−1^ in the spectra of products was assigned to M-O group [14] as in Figures 2 and 3.  Table 2 shows the IR spectra of ligands and their complexes experimentally and theoretically using semiempirical (PM6) method.

### 3.3. UV-Visible Spectra

**Figure 4:**
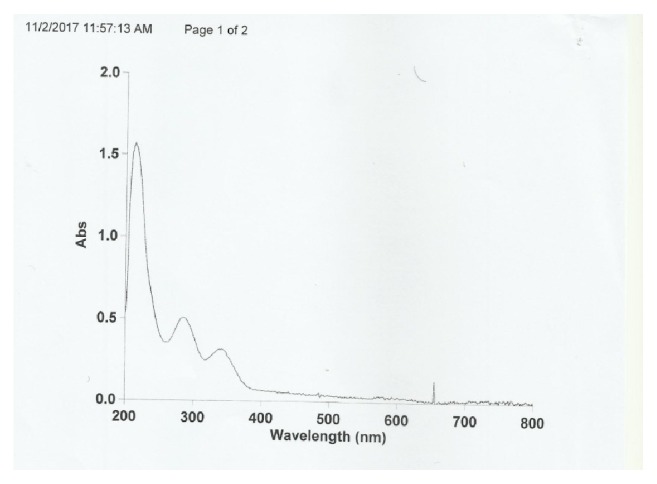
UV-Vis spectra of the complex Co(L_1_)(L_2_) H_2_OCl].

The electronic spectra of the ligands L_1_, L_2_ and their metal complexes were measured in ethanol and DMSO solutions. The mefenamic acid and metformin base ligand L_1_, L_2_ displayed absorption around and in the 220 nm region assigned to the *π* → *π∗* transition that is unaffected in the formation of complexes. The peaks around 290 and 350 nm are assigned to n → *π∗* transitions of (-C=N-, -C=C, C=O) groups and intraligand charge transfer [15]. Transition metal complexes showed the following *d* → *d* transition. Two bands observed for Co^+2^ complex go to the transitions ^4^T_1_g→^4^T_2_g(F) and ^4^T_1_g(F)→^4^T_1_g(P), respectively, suggesting high spin octahedral geometry (t_2_g^5^eg^2^) [16]. For Cu^+2^ complex, only two broad bands were recorded for the first at 16149cm^−1^ and shoulder for the second at 27027cm^−1^, while the third one did not appear due to its position at infrared region suggesting a distorted octahedral geometry [17].  Figure 4 and Table 3 show the UV-Vis spectra of the complex [Co(L_1_)(L_2_)H_2_OCl].

### 3.4. Thermogravimetric Analysis (TGA)

**Figure 5:**
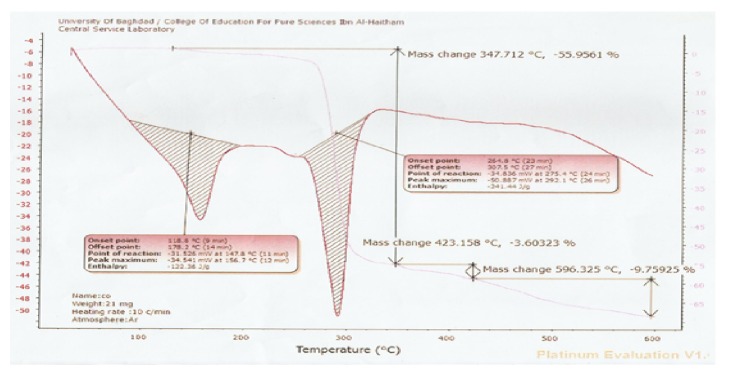
TG/DTG and DSC thermogram of [Co(L^1^)(L^2^) H_2_OCl] in argon atmosphere.

**Figure 6:**
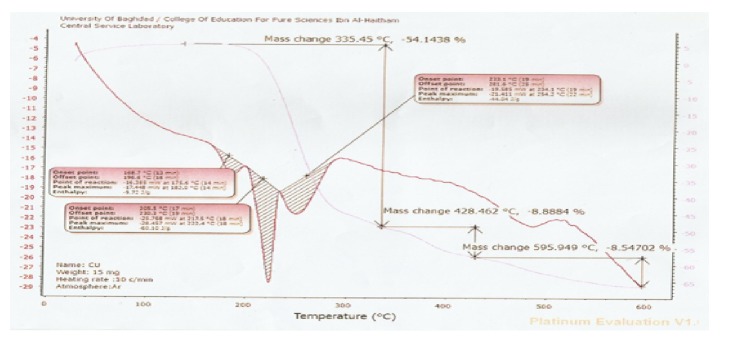
TG/DTG and DSC thermogram of [Cu(L^1^)(L^2^) H_2_OCl] in argon atmosphere biological activity.

Thermogravimetric analyses (weight changes) were performed in the temperature up to 600°C under argon atmosphere at the heating rate 20 C/min. The thermogram of Co^II^, Cu^II^ complexes recorded three stages of weight loss as shown in Figures 5 and 6. The first one showed the initial weight loss in the temperature around 347, 335 C probably be due to the loss in the coordinated big weight due to the decomposition of organic constituents of complexes molecule. The metal oxide could represent the final residue with attaining a constant weight [18]. Furthermore the DSC analysis of nickel (II) complexes showed the stability of complexes in inert helium gas and the peaks, being exothermic, were very important to estimate some thermodynamic terms like enthalpy, entropy, and Gibbs-Free energy as shown in Figures 5 and 6.

### 3.5. Biological Activity

**Figure 7:**
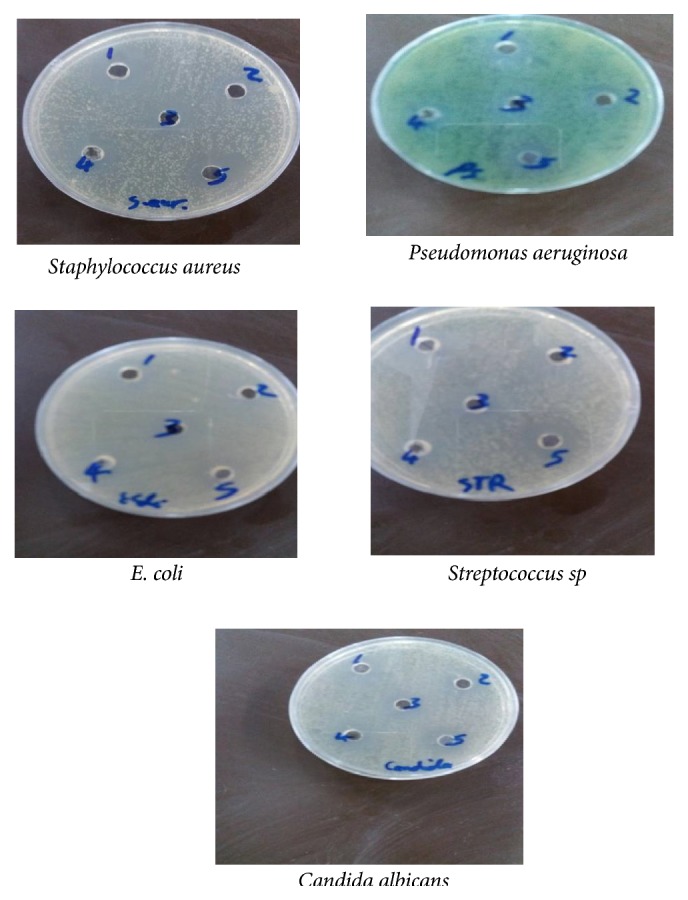
Effect of ligands and their complexes in the inhibition of different kinds of bacteria.

Biological activities of these complexes against different bacterial isolates were studied. The biological activities of the test compounds were evaluated by the well diffusion method against* E. coli, Staphylococcus aureus, Streptococcus mutans*,* Pseudomonas aeruginosa*,* and Candida albicans *(fungus). In this method pure isolate of 24hr growth was cultured in Muller-Hinton Agar plate (Hi Media, Mumbai, India) by using sterile swab so as to achieve a confluent growth. The plates were allowed to dry and a sterile cork borer of diameter 8.0mm was used to bore four wells in each agar plates. A 10*μ*L volume of each complex was applied by micropipette in the wells into Muller-Hinton Agar plate. Distilled water served as control. The plates were allowed to stand for 1h or more for diffusion to take place and then incubated at 37°C for 24hrs. The zone of inhibition was recorded [19]. Several researches have shown that coordination of organic compounds to a metallic element causes significant changes in the biological activity of both the organic ligand and the metal. The ligands showed antimicrobial activity against both kinds of bacteria. The complex Co-ligand 1/ligand 2/DMSO has the highest inhibition zone among the others. The complex Cu- ligand 1/ligand 2/DMSO also showed significant antimicrobial activity effect, which does not have antibacterial activity against* Staphylococcus aureus* and* Streptococcus mutans* shown in the higher inhibition zone of metal complexes than those of the ligands, which can be explained on the basis of Overtone's concept and chelation theory. On chelation, the polarity of the metal ion will be reduced to a greater extent due to the overlap of the ligand orbital and partial sharing of the positive charge of the metal ion with donor groups. The weak antibacterial activity for Cu-ligand/DMSO and Co-ligand/DMSO against gram negative bacteria was ascribed to the presence of an outer membrane, which poses hydrophilic polysaccharides chains as a barrier to these complexes. Cobalt is not generally considered to be a very toxic element [20, 21]. A large number of reports on the antibacterial properties of cobalt complexes have shown Co(II) complexes to be the most studied probably due to their aqueous stability, accessibility, and ease of synthesis. However, only a small number of cobalt (III) complexes have biochemical roles. Vitamin* B12 *is a cobaloxime, a cobalt complex having a glyoxime ligand, and is one of the unusual examples of a naturally occurring organometallic complex, i.e., possessing a metal carbon bond [22].  Figure 7 and Table 4 show the effect of ligands and their complexes on different kinds of bacteria.

## 4. Conclusions

We concluded in this work that the synthesized complexes, prepared from mixed drugs (mefenamic acid and metformin), had octahedral geometric shapes; the biological activity of these drugs against different kinds of bacteria increased after being mixed with Cu(II) and Co(II). We concluded that there is a great convergence between experimental and theoretical results using semiempirical (PM6) method.

## Figures and Tables

**Table 1:** Physicochemical properties of ligands and synthesized complexes.

Comp.	M.Wt	Color	Melting Point (°C)	Yield(%)	Molar Conductance (ohm^−1^.mol^−1^.cm^2^)	B.M(*μ*)
C15H15NO2 [L_1_]	241	White	230-231	- - - - - -	- - - - - - -	- - - -
C4H11N5 [L_2_]	129	White	221-222	- - - - - -	- - - - - - -	- - - - -
[Co(L_1_)(L_2_) H_2_OCl]	481	Violet	>300	71	18	4.7
[Cu(L_1_)(L_2_) H_2_OCl]	486	Pink	>300	83	11	1.65

**Table 2:** Experimental and theoretical IR spectrum bands of ligands and their complexes.

Comp.	*υ*(N-H)	*υ*(C=O)	*υ*(COO)	*υ*(M-O)
C15H15NO2 [L_1_]	3340	1730	1673-1422	- - - - - -
	(3332)_P_	(1721)_P_	(1676-1396)_P_	
C4H11N5 [L_2_]	3373	- -	- - - -	- - - - -
	(3388)_P_			
[Co(L_1_)(L_2_) H_2_OCl]	3327	- - -	1678-1492	486
	(3315)_P_		(1672-1494)_P_	(490)_P_
[Cu(L_1_)(L_2_) H_2_OCl]	3336	- - - - -	1614-1462	491
	(3334)_P_		(1615-1458)_P_	(499)_P_

^*∗*^P: semiempirical (PM6) method.

**Table 3:** Experimental and theoretical UV-Vis spectrum bands of ligands and their complexes.

Compounds	Bands (nm)	Geometry
C15H15NO2 [L_1_]	324 – 247	- - -
	(323-237)_P_	
C4H11N5 [L_2_]	237 – 266	- - - -
	(222 – 265)_P_	
[Co(L_1_)(L_2_) H_2_OCl]	610 – 489 - 341	Octahedral
	(596 – 484 – 337)_P_	
[Cu(L_1_)(L_2_) H_2_OCl]	580 – 370 - 290	Octahedral
	(670 – 365 – 296)_P_	

^*∗*^P: semiempirical (PM6) method.

**Table 4:** Biological activities of the ligands and their complexes against different bacterial isolates.

Bacterial isolates	DMSO	Ligand 1∖DMSO	Ligand 2/DMSO	Ligand	Co ligand	Cu-ligand
				1+2/DMSO	1+2/DMSO	1+2/DMSO
*Staphylococcus aureus*	-	21	15	14	14	15
*Pseudomonas aeruginosa*	-	14	-	-	13	10
*E. coli*	-	15	-	-	15	12
*Streptococcus sp.*	-	15	-	12	15	11
*Candida albicans*	-	14	14	12	16	13

## Data Availability

The data used to support the findings of this study are available from the corresponding author upon request.
